# The mysterious case of the disappearing pilot study: a review of publication bias in preliminary behavioral interventions presented at health behavior conferences

**DOI:** 10.1186/s40814-023-01345-8

**Published:** 2023-07-07

**Authors:** Lauren von Klinggraeff, Kaitlyn Ramey, Christopher D. Pfledderer, Sarah Burkart, Bridget Armstrong, R. Glenn Weaver, Michael W. Beets

**Affiliations:** Department of Exercise Science, Arnold School of Public Health, University of South Carolina, 921 Assembly Street, Columbia, SC USA

**Keywords:** Pilot projects, Feasibility studies, Publication bias, Gray literature, Peer review, Research

## Abstract

**Background:**

The number of preliminary studies conducted and published has increased in recent years. However, there are likely many preliminary studies that go unpublished because preliminary studies are typically small and may not be perceived as methodologically rigorous. The extent of publication bias within preliminary studies is unknown but can prove useful to determine whether preliminary studies appearing in peer-reviewed journals are fundamentally different than those that are unpublished. The purpose of this study was to identify characteristics associated with publication in a sample of abstracts of preliminary studies of behavioral interventions presented at conferences.

**Methods:**

Abstract supplements from two primary outlets for behavioral intervention research (Society of Behavioral Medicine and International Society of Behavioral Nutrition and Physical Activity) were searched to identify all abstracts reporting findings of behavioral interventions from preliminary studies. Study characteristics were extracted from the abstracts including year presented, sample size, design, and statistical significance. To determine if abstracts had a matching peer-reviewed publication, a search of authors’ curriculum vitae and research databases was conducted. Iterative logistic regression models were used to estimate odds of abstract publication. Authors with unpublished preliminary studies were surveyed to identify reasons for nonpublication.

**Results:**

Across conferences, a total of 18,961 abstracts were presented. Of these, 791 were preliminary behavioral interventions, of which 49% (388) were published in a peer-reviewed journal. For models with main effects only, preliminary studies with sample sizes greater than *n* = 24 were more likely to be published (range of odds ratios, 1.82 to 2.01). For models including interactions among study characteristics, no significant associations were found. Authors of unpublished preliminary studies indicated small sample sizes and being underpowered to detect effects as barriers to attempting publication.

**Conclusions:**

Half of preliminary studies presented at conferences go unpublished, but published preliminary studies appearing in peer-reviewed literature are not systematically different from those that remain unpublished. Without publication, it is difficult to assess the quality of information regarding the early-stage development of interventions. This inaccessibility inhibits our ability to learn from the progression of preliminary studies.

**Supplementary Information:**

The online version contains supplementary material available at 10.1186/s40814-023-01345-8.

## Key messages

- Little is known about how publication bias may affect preliminary or feasibility studies in behavioral studies, but studies of publication bias indicate smaller, statistically nonsignificant studies are less likely to be published.
- About half of all preliminary behavioral interventions go unpublished, and those appearing in the published literature are not functionally different than those that go unpublished.
- Researchers conducting preliminary behavioral interventions should consider sharing unpublished findings on open-source platforms to maximize the fields’ ability to learn from their work.

## Introduction

Publication bias is a phenomenon in which the characteristics and outcomes of a research study impact the likelihood of publication [1]. It is well-documented that small and non-statistically significant studies are less likely to appear in a peer-reviewed journal and hence are subject to publication bias [2]. Preliminary studies, which are those studies conducted to establish the feasibility and acceptability of a behavioral intervention [3], are often characterized as being small and underpowered to detect statistically significant findings [4, 5]. Since preliminary studies commonly have features associated with publication bias, they may be substantially underrepresented in the published literature.

Publishing preliminary studies is important for multiple reasons. Publication provides detailed information that serves as a foundation for subsequent larger-scale trials, which the preliminary studies are designed to inform. Publication also disseminates useful information to the research community about what may or may not work regarding the development of new or adapted intervention approaches [6–8]. Preliminary studies of behavioral interventions often appear in the gray literature, such as conference abstracts. This is due to the lower barriers to entry when compared to publication in peer-reviewed journals [9]. Using conference abstracts to gauge the extent of publication bias has been used as a strategy to explore publication bias in medical fields [9], though less is known about the impact of publication bias of preliminary studies in the behavioral intervention field.

The objectives of this study are as follows:

- Objective 1: Identify the prevalence of publication bias among preliminary studies presented at conferences on behavioral health interventions.
- Objective 2: Examine study characteristics associated with full-length publication in peer-reviewed journals among preliminary studies presented at conferences on behavioral health interventions.
- Objective 3: Identify potential reasons preliminary studies presented at conferences on behavioral health interventions may go unpublished.

## Methods

### Objective 1

Identify the prevalence of publication bias among preliminary studies presented at conferences on behavioral health interventions.

To identify the prevalence of publication bias among preliminary studies presented at behavioral health conferences, we created a dataset containing preliminary studies presented at conferences on behavioral health interventions. First, we identifed conferences presenting behavioral interventions at the following conferences: (1) the Society of Behavioral Medicine (SBM), (2) International Society of Behavioral Medicine (ISBNPA), (3) American Public Health Association (APHA) Annual Conference, and (4) Society for Prevention Research (SPR). Second, we attempted to access all meeting supplements from those conferences. The (1) SBM Annual Meeting Supplements published from 2006 to 2017 were downloaded via the *Annals of Behavioral Medicine* published by Oxford Academic. (2) Official electronic booklets containing the abstracts presented at ISBNPA for 2009–2010 and 2012–2017 were obtained via email from conference organizers. No booklet for the ISBNPA 2011 Annual Meeting was available, and only abstracts of poster presentations were available for the 2012 and 2016 ISBNPA conferences. (3) Six attempts were made to obtain records of conference abstracts from the annual meeting of SPR, but no response was received. (4) Abstracts presented at the APHA were also considered for this study, but based on preliminary data, the undertaking was determined to be too resource intensive (73,511 estimated abstracts with preliminary terms).

Next, SBM and ISBNPA electronic conference booklets/supplements were uploaded into NVivo (QSR International, Doncaster, Australia) and searched via the text search query, using the keywords “preliminary,” “feasibility,” “pilot,” or “exploratory” to identify preliminary studies. After abstracts were flagged in NVivo as containing a keyword, each was read by the first author (L. V.) to determine if it met the eligibility criteria for a behavioral intervention. Abstracts determined to be preliminary studies meeting the inclusion criteria were retained to form a dataset containing preliminary studies presented at conferences on behavioral health interventions.

#### Inclusion/exclusion criteria

Preliminary behavioral interventions were defined as studies designed to test the feasibility of a behavioral intervention and/or provide evidence of preliminary effect(s) [10]. Conference abstracts which reported preliminary protocols, mechanistic studies conducted in laboratories, and scale, tool (i.e., exploratory factor analysis), or device development were not included. Pharmaceutical and surgical trials were excluded. Isolated arms of multi-arm trials were also excluded.

In order to identify the prevalence of publication bias among preliminary studies presented at behavioral health conferences, we next searched for publications resulting from the presented abstracts (herein referred to as “publications”). To do this, publication databases (PubMed/MEDLINE, Web of Science, Embase, EBSCOhost), author’s curriculum vitae, ResearchGate (https://www.researchgate.net), ORCID (https://www.orcid.org), and Google Scholar (https://scholar.google.com) were manually searched using the title, body, and author list of each conference abstract in order to find a publication that met the pairing criteria.

#### Pairing criteria

Publications were considered to “match” conference abstracts if the publication reported identical intervention components including duration, location, and target population, though they could vary on reported sample size by + / − 5 participants. Additionally, matched abstracts and publications were required to share at least one author with the abstract (often the first or lead/final author). Where conference abstracts did not present the necessary information needed for pairing (e.g., did not present sample size), efforts were made to pair the conference abstract with similar publications from the first or corresponding authors. Publications were required to be full-length original research articles in peer-reviewed journals. Ulrichsweb (www.ulrichsweb.serialssolutions.com) was used to determine if journals were peer reviewed. When multiple conference abstracts were matched to a single publication, each abstract-to-publication pair was counted as an individual pair. When likely parings were found, authors were emailed for confirmation. Pairings were made by two members of the research team (L. V., K. R.) and then verified by additional members of the research team (S. B., M. B.).

### Objective 2

Examine study characteristics associated with full-length publication in peer-reviewed journals among preliminary studies presented at conferences on behavioral health interventions.

To examine study characteristics associated with full-length publication in peer-reviewed journals among preliminary studies presented at behavioral health conferences, we added additional information to our dataset about relevant features of the abstracts. This information was extracted from the conference abstracts rather than publications because publications were not available for all abstracts (i.e., the unpublished studies). Data extraction was completed in Microsoft Excel 365 (Microsoft, Redmond, WA, USA) by two members of the research team (L. V., K. R.). Where information was provided, conference abstracts were coded as follows: (a) conference abstract characteristics (year of conference, associated society); (b) study characteristics (methodology, sample size, randomization, control/comparison or similar additional group, mention of significant or positive findings, claims that future work was warranted, inferential statistical significance); (c) institutional affiliation of the first author; (d) location of authors’ institution (i.e., country); and (e) abstracts’ description of participants.

Next, we used random-effects logistic regression models to assess the odds that an abstract would be published as a full-length article [9]. An iterative model building approach was utilized [8, 11] which accounted for the following covariates and control variables commonly reported in conference abstracts: (1) conference association (i.e., SBM, ISBNPA); (2) year of conference abstract presentation (i.e., continuous variable for year); (3) statistical significance as presented in conference abstract (i.e., binary presence/absence); (4) sample size category with samples less than or equal to 24 participants as the referent group (i.e., ≤ 24, 25–49, 50–99, ≥ 100, unspecified sample size); and (5) randomized multi-arm design (i.e., binary presence/absence). Subsequent models accounted for two-way and three-way interactions between covariates. All analyses had an alpha level of 0.05 and were carried out using STATA v16.0 statistical software package (College Station, TX, USA). Conference abstract methodology was categorized into three types (quantitative, qualitative, mixed methods), and models were run on quantitative studies only.

### Objective 3

Identify potential reasons preliminary studies presented at conferences on behavioral health interventions may go unpublished.

After analyses were conducted on the dataset described above, we sought to identify potential reasons preliminary studies presented at conferences on behavioral health interventions may go unpublished. Using Qualtrics (Qualtrics International, Seattle, WA, USA), emails were sent to the first or corresponding author for conference abstracts that had not been paired to full-length peer-reviewed journals. ISBNPA official electronic booklets did not provide authors’ emails; therefore, only SBM authors were surveyed. If an author’s email could not be delivered to a recipient’s inbox, two additional attempts were made to identify current email addresses for the first or corresponding authors. The survey was first distributed on October 14, 2021, and was closed on January 11, 2022. Respondents were provided with an electronic US $25 Amazon gift card as compensation. Approval from the institutional review board was obtained prior to survey distribution (Pro00116014).

The survey was designed to (1) assess the accuracy of the matching procedures to ensure it adequately captured published preliminary studies and (2) to understand reasons preliminary studies may go unpublished. Authors were provided with their SBM conference abstracts’ title and year of presentation and asked to provide a citation or digital object index (DOI) if that study had been published. If authors confirmed that their study had not been published, they were asked to select from provided reasons for nonpublication including resources (e.g., lack of money for publication fees, lack of administrative support), study characteristics (e.g., null results), external factors (e.g., difficulties with co-authors), and features common to preliminary studies (e.g., small sample size) and provided with open-response opportunities to provide additional information. The full survey can be found in Additional file 1: Appendix 1. If surveyed authors provided citations or DOI to published studies, the first author (L. V.) compared the provided publication to the conference abstract to verify that the conference abstract and publication met matching criteria. Survey responses from authors of unpublished preliminary studies were analyzed descriptively in STATA v16.0 (StataCorp, College Station, TX, USA).

## Results

### Objective 1

Identify the prevalence of publication bias among preliminary studies presented at conferences on behavioral health interventions.

Across SBM and ISBNPA, 12,915 and 6046 conference abstracts were obtained, respectively, from available records, for a total of 18,961 conference abstracts. A total of 5323 conference abstracts were identified as containing preliminary-related terms (3546 SBM; 1777 ISBNPA). Upon review, 791 were identified as preliminary behavioral interventions meeting the pre-specified inclusion criteria (484 SBM; 307 ISBNPA). This decrease in the number of abstracts between the keyword count and those retained in the dataset was due to the frequent use of preliminary keywords in measurement studies and mechanistic studies. For example, the keyword “exploratory” often returns abstracts containing “exploratory factor analysis.” Similarly, studies may “pilot a survey” or test the “feasibility” (i.e., tolerability) of mechanistic interventions (e.g., controlled light exposure, feeding study). These studies are not considered behavioral interventions and were excluded. Of the identifed 791 abstracts, 388 were published in peer-reviewed journals for an overall publication rate of 49.1% across both conferences (224 SBM; 164 ISBNPA; Table 1).

**Table 1:** Summary of research abstracts presented at the Society of Behavioral Medicine (SBM) and International Society of Behavioral Nutrition and Physical Activity (ISBNPA) from 2006 to 2018

	SBM					ISBNPA				
Year^a^	Total abstracts (*n*)	Pilot studies (*n*)	Published pilot studies (*n*)	% pilot studies^b^	% pilot studies published^c^	Total abstracts (*n*)	Pilot studies (*n*)	Published pilot studies (*n*)	% pilot studies^b^	% pilot studies published^c^
2006	747	13	7	2%	54%	-	-	-	-	-
2007	861	25	11	3%	44%	-	-	-	-	-
2008	904	26	9	3%	35%	-	-	-	-	-
2009	935	31	15	3%	48%	607	12	5	2%	42%
2010	858	21	10	2%	48%	492	15	14	3%	93%
2011	1023	31	15	3%	48%	-	-	-	-	-
2012	1118	38	18	3%	47%	239^d^	14	7	-	50%
2013	1289	36	16	3%	44%	856	35	22	4%	63%
2014	1118	59	34	5%	58%	870	45	21	5%	47%
2015	1011	56	23	6%	41%	1290	54	31	4%	57%
2016	1317	71	41	5%	58%	658^d^	28	16	-	57%
2017	1734	77	25	4%	32%	1034	59	32	6%	54%
2018	-	-	-	-	-	777	45	16	6%	36%
Overall	12,915	484	224	4%	46%	5926	307	164	5%	53%

^a^Year represents the year the conference was held

^b^Percent pilot studies indicate all conference abstracts within the specified year that report pilot behavioral interventions

^c^Percent pilot studies published indicate all conference abstracts reporting pilot behavioral interventions within the specified year that are subsequently published at full length in a peer-reviewed journal

^d^Denotes years when only records of poster presentation abstracts were accessible (i.e., abstracts of oral presentations were not available)

### Objective 2

Examine study characteristics associated with full-length publication in peer-reviewed journals among preliminary studies.

The odds of subsequent publication among quantitative preliminary studies are reported in Table 2; additional abstract characteristics are presented in Table 3. Iterative models indicated that several factors were initially associated with increased odds of full-length publication including RCT design and larger sample sizes. However, these effects were null in models accounting for interactions between study design, sample size, and study significance.

**Table 2:** Summary of iterative models predicting odds of publication based on abstracts characteristics

Variable	Model 1			Model 2			Model 3			Model 4			Model 5		
	OR	95% *CI*		OR	95% *CI*		OR	95% *CI*		OR	95% *CI*		OR	95% *CI*	
Randomized multi-arm trial	**1.52**	**1.09**	**2.11**	**1.49**	**1.07**	**2.08**	1.30	0.92	1.85	0.96	0.57	1.62	0.77	0.21	2.82
Significance				1.17	0.81	1.70	1.12	0.76	1.66	1.03	0.56	1.92	0.86	0.37	1.99
Sample size (*n* ≤ 24 referent)															
25–49							**1.89**	**1.19**	**3.02**	**1.88**	**1.18**	**2.99**	1.96	0.63	6.16
50–99							**2.01**	**1.25**	**3.25**	**2.00**	**1.24**	**3.23**	1.10	0.36	3.39
≥ 100							**1.82**	**1.09**	**3.03**	**1.81**	**1.08**	**3.01**	1.01	0.34	2.97
Randomized multi-arm trial × significance										1.40	0.67	2.95	1.01	0.22	4.75
Randomized multi-arm trial × sample size															
25–49													0.90	0.14	5.88
50–99													2.22	0.38	13.06
≥ 100													1.66	0.28	9.75
Significance × sample size															
25–49													0.86	0.22	3.38
50–99													0.96	0.23	3.93
≥ 100													1.84	0.46	7.41
Randomized multi-arm trial × significance × sample size															
25–49													1.87	0.21	16.89
50–99													1.87	0.22	15.94
≥ 100													1.07	0.12	9.67
Conference^a^	0.73	0.51	1.05	0.71	0.49	1.03	0.78	0.53	1.15	0.77	0.52	1.14	0.73	0.49	1.08
Year^b^	1.04	0.98	1.09	1.03	0.98	1.09	1.04	0.98	1.10	1.04	0.98	1.10	1.04	0.99	1.11

**Bold** text indicates statistically significant, *p* ≤ 0.05

^a^ISBNPA referent

^b^Year label represents year of abstract presentation

**Table 3:** Summary of abstract characteristics with percentage of abstracts (*n* = 791) paired to full-length, peer-reviewed articles^a^

	**Abstracts**	**Published**
	*n*	*n* (%)
**Methodology**		
Quantitative	578	283 (49%)
Mixed	167	90 (54%)
Qualitative	28	9 (32%)
Unspecified	18	6 (33%)
**Affiliation of authors’ institution**^b^		
Government	25	8 (68%)
Independent	22	7 (68%)
Medical	30	12 (60%)
University	407	197 (52%)
**Country of authors’ institution**^c^		
High income	776	384 (49%)
Upper middle income	11	2 (18%)
High & upper middle income	2	1 (50%)
Lower middle income	1	0 (0%)
High & low income	1	0 (0%)
**Participant demographic**^d^		
Adult	280	147 (53%)
Unspecified	212	101 (48%)
Older adult	83	46 (55%)
Children	80	41 (51%)
Adolescence	59	26 (44%)
Young adult	35	10 (29%)
Family	19	4 (21%)
Couple or dyad	12	5 (42%)
Site	6	4 (67%)
Infant	5	4 (80%)

^a^Percentages may not add to 100% due to rounding

^b^Available only for abstracts presented at the Society of Behavioral Medicine (*n* = 484)

^c^Categories determined using the World Bank country classifications for the 2023 fiscal year. Multiple classifications are listed where abstract authors were from more than one classification

^d^Demographics were specified by abstracts. Abstracts not specifying a target population were coded as “unspecified”

The year of conference was a control variable in models predicting odds of publication and was not statistically significant, though there was a consistent positive trend in the number and proportion of preliminary behavioral intervention conference abstracts presented at SBM and ISBNPA across time (Table 1). For example, in 2009, 13 preliminary behavioral interventions were presented at SBM, constituting 2% of all conference abstracts presented that year. In 2017, 77 preliminary behavioral intervention abstracts were presented at SBM, constituting 4% of all the conference’s abstracts that year. Of similar interest, we descriptively explored how the year of publication was related to the year of conference presentation. Publication typically occurred after conference presentation (*n* = 245, 63%). Fewer conference abstracts were published during the same year of the conference presentation (*n* = 108, 27%) or before (*n* = 40, 10%). Notably, five conference abstracts were published more than 2 years prior to their presentation at conferences.

Sample size was predictive of publication in models 3 and 4, but not in models where interactions between sample size and other predictor variables were included. Descriptively, a total of 754 (95%) conference abstracts provided the sample size of their study. The mean number of participants across all reporting conference abstracts was 101 (*SD* 253) with a range of 2 to 3147 (median 40, *IQR* 20–78).

Randomized multi-arm designs were predictive of publication in models 1 & 2, but not in models accounting for sample size.

Neither inferential claims of statistical significance or positive change and conference (i.e., SBM, ISBNPA) nor claims of future work were associated with increased odds of publication in any model. A total of 394 (68%) quantitative conference abstracts presented inferential claims of statistical significance or positive change (e.g., hypothesis confirming), using phrases such as “significantly different,” “positive change from baseline,” or “clinically significant.” Most conference abstracts (*n* = 547, 71%) indicated further work was warranted given preliminary study results, and 22 conference abstracts (3%) indicated a current subsequent study was underway at the time of conference abstract presentation.

### Objective 3

Identify potential reasons preliminary studies presented at conferences on behavioral health interventions may go unpublished.

**Fig. 1:**
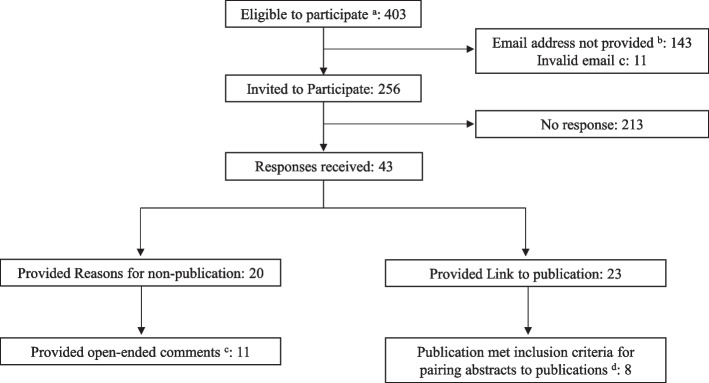
A CROSS flow diagram [13] of survey respondents initially identified as having unpublished preliminary behavioral interventions. ^a^Eligible respondents were those identified as having presented a preliminary behavioral intervention at the Society of Behavioral Medicine (SBM) or International Society for Behavioral Nutrition and Physical Activity (ISBNPA), but which had not been identified as having full-length, peer-reviewed articles. ^b^Email addresses for authors were not provided in ISBNPA records. ^c^Open-ended comments were those provided in free-text or to open-ended questions in the survey. Answering these questions was optional and not required to complete the survey. A list of the 11 responses is provided in Additional file 2: Appendix 2. ^d^Consistent with prior research, paired preliminary studies were defined as full-length original research articles in peer-reviewed journals. They also were required to report identical intervention components including duration, location, and target population and could not vary on reported sample size by greater than ± 5 participants

Out of the 484 SBM abstracts, 267 were unpublished, 256 working emails were identified for first or corresponding authors, and 43 responded (17% response rate; Fig. 1). This is a lower response rate compared to response rates reported in a review of surveys asking author about non-publication (66% (*IQR*: 50 to 80%) [12]. The respondents were 72% female with 65% between ages 35 and 50. Of the 43 respondents, 23 (54%) provided citations or DOI links for published studies, and the remaining 20 (47%) provided reasons for non-publication; 11 provided open-ended responses in addition to answering multiple-choice questions. Among those providing citations/DOI links, 15 did not meet our prespecified pairing criteria. Specifically, 4 were not published in refereed journals, 7 presented different sample sizes between the abstract and full-length publication exceeding + / − 5 participants, and 4 reported single arms of multiarmed studies. Of the 8 studies which were accurate pairings, 2 had not initially been identified because they were published after the research team finished searching for subsequent full-length publications (e.g., 2020 onward), and 6 studies were initially missed during pairing. These 6 were accounted for in the final dataset and used in the presented analysis and tables. Of the 20 authors who did not publish their study, 16 initially intended to publish their study, 4 authors submitted their study to a journal, and one author submitted to more than two journals.

Small sample size was the most common reason for non-publication of preliminary studies. Open-ended responses specifically mentioned low power, being underpowered to detect efficacy, and extremely small sample sizes (Q2.12; Additional file 2: Appendix 2). Small sample size was also the most commonly selected multiple-choice answer (*n* = 11) when asked about common pilot features that inhibit publication (Q2.12; Additional file 1: Appendix 1). Capacity for publishing, specifically related to the composition of the research team, was mentioned by 7 respondents. This included remarks that the students led the writing, research staff turnover was high, principal investigators changed institutions, lack of project coordinators resulting in diminished time for writing publications, or lack of expertise or mentored support (Q2.20 & Q2.13; Additional file 2: Appendix 2). In multiple-choice responses, 8 respondents mentioned lack of time (Q2.14; Additional file 1: Appendix 1), and 9 stated that other projects had taken away their focus on publishing the preliminary study (Q2.13; Additional file 1: Appendix 1).

Most respondents (*n* = 16) thought publishing preliminary studies was somewhat or extremely difficult (Q2.17; Additional file 1: Appendix 1). Authors indicated that they had published other preliminary studies using strategies such as submitting to journals focused on preliminary work or putting findings into letters to the editor and brief reports rather than in full-length publications (Q2.20; Additional file 2: Appendix 2). When asked about alternative publishing platforms such as ResearchGate, Octopus, and Open Science, 70% (*n* = 14) were aware they existed (Q2.18; Additional file 1: Appendix 1), and 35% (*n* = 7) had used them to share research (Q2.19; Additional file 1: Appendix 1).

## Discussion

In our first and second objectives, we sought to examine the prevalence and potential predictive characteristics of publication bias in preliminary behavioral interventions (e.g., pilot, feasibility studies). Our results suggest preliminary studies with sample sizes of at least twenty-five participants are at increased odds of publication. No other extracted study characteristics predicted publication. Surprisingly, statistical significance was not related to subsequent full-length publication in a refereed journal. This contrasts typical patterns of publication bias wherein trials with statistically significance effects are at increased odds of publication [9, 14–16]. Ultimately, we did not find evidence to suggest the preliminary behavioral interventions appearing in the published literature are functionally different than those that go unpublished.

Consistent with other studies of publication bias in clinical and health services research [9, 12, 17–19], approximately half of all preliminary behavioral interventions presented at conference mysteriously “disappear” before they reach the published literature. These disappearances limit our knowledge of what has already been tested and cut short our ability to learn from the progression of behavioral interventions. Preliminary studies play a crucial role in the development of behavioral interventions [20–23], providing valuable information regarding intervention viability for larger-scale testing. Without access to publications, information regarding the early-stage development of interventions including information on trial feasibility (e.g., successful measurement collection), intervention feasibility (e.g., participant acceptability), and implementation is inaccessible to grant reviewers and other researchers in similar fields. This inaccessibility results in research waste [24], inhibits the speed of intervention development to address pressing public health needs [25], inhibits our ability to learn from the progression of preliminary studies [6, 10], and is inconsistent with good scientific practice [7, 26].

For our third objective, we sought to identify potential reasons preliminary studies may go unpublished. It seems that preliminary studies may remain unpublished because authors believe preliminary studies must contain evidence of efficacy, or at least the sample size to test for efficacy, to be accepted for publication. Though recommendations call for the reporting of trial and study feasibility rather than efficacy [27, 28], the perception remains that smaller efficacy studies and those not reporting efficacy are unlikely to be published [29]. Authors are unlikely to prioritize submitting preliminary studies for publication given the perception that the paper may never be accepted for publication and therefore be a poor use of time. Of the authors surveyed, 80% intended to publish their pilot study, but only 25% submitted their study for publication even once. This indicates that the primary impediment to publishing preliminary studies occurs after studies are completed but before they are submitted for publication.

### Implications

We suggest authors of preliminary studies seek to publish their studies in peer-reviewed journals and, if not successful, seek alternative platforms for sharing their findings to reduce publication barriers. Preprint servers such as ResearchGate, Open Science Framework, medrXiv, aRxiv, and bioRxiv can serve as platforms outside of traditional scientific journal publication which can reduce the reported lack of time and difficulty of publishing. These interfaces move more quickly than traditional publishing outlets which take time for reviews, editorial correspondence, and copy editing. Arguments may remain that non-indexed research outlets lack incentivization in the traditional tenure and promotion trajectory and therein may fail to solve the reported issue of low priority for publishing preliminary studies. However, it is our belief that if journal editors and funders of larger trials (e.g., National Institutes of Health [NIH]) required access to full reports of preliminary studies during the review and publication of subsequent related larger trials, the number of accessible preliminary studies would increase.

### Limitations

Based upon previous literature, randomization and control-group presence were hypothesized to be strong predictors of subsequent publication [9, 30], and due to this consideration, only conference abstracts with quantitative methodology were included in model estimations. Conference abstracts which did not contain sample size (*n* = 37, 5%), and those not containing information sufficient to determine methodology (*n* = 18, 2%), were not included in the models because this lack of information made it difficult to pair with subsequent publication, and their inclusion could have inflated estimates of non-publication.

Not all possible publication types are accounted for in this study. Inclusion criteria based on previous literature required that pairs be made between abstracts and full-length peer-reviewed journal articles. This may have excluded non-peer-reviewed publications, government, nongovernmental organizations, or foundational/philanthropic reports. This choice was made to prioritize indexed, accessible knowledge that would allow potential grant reviewers and intervention scientists access to detailed information about preliminary studies. Additionally, factors outside of study specific characteristics may drive publication odds, and our study did not account for covariates such as author gender, institution ranking, author recognition, funding, author career rank, or conference presentation format (e.g., poster or oral presentation). Efforts were made to acquire this information, but complete, accurate data could not be obtained. Additionally, we cannot know whether there is initial bias in the preliminary studies submitted, accepted, and presented at conferences and admit that our sample of conference abstracts presenting preliminary behavioral interventions is incomplete. Abstracts reporting preliminary work are presented at many outlets including internal university symposia and regional conferences. We did make efforts to expand our sample through APHA and SPR though these were either untenable or unattainable. Ultimately, our picture of the total scope of preliminary work is incomplete.

## Conclusion

Preliminary studies play a key role in the development of behavioral interventions by providing information about the practicality, execution, and resources needed to execute a larger iteration of the same or similar study [31, 32]. However, the present study found that nearly 50% of preliminary behavioral interventions presented at conferences go unpublished. Institutions such as the NIH and Medical Research Council of the UK increasingly require preliminary data in grant applications and have designed specific funding mechanisms to support their implementation (e.g., NIH R34). Grant reviewers within these institutions rely on details of preliminary trial feasibility, trial parameters, and participant acceptability to gauge a proposal’s odds of success in a larger trial. Additionally, translational research and intervention scientists look to previously conducted preliminary studies to improve the efficiency and effectiveness of future interventions [6, 10]. Given the incomplete rate of preliminary study publication, and the commonly reported barriers to publication, presenting preliminary study findings on searchable, open-source platforms may increase the amount of information available to funding review panels and scientists, maximizing the fields’ ability to invest in and design high-quality, scalable behavioral health interventions for population health.

## Supplementary Information

**Additional file 1: Appendix 1.** Survey Questions.

**Additional file 2: Appendix 2.** Open Ended Responses.

**Additional file 3: Appendix 3.** Checklist for Reporting Of Survey Studies (CROSS).

## Data Availability

Preliminary data from this study were presented at the 19th Annual Meeting of the International Society of Behavioral Nutrition and Physical Activity (2020) and the 41st Annual Meeting of the Society of Behavioral Medicine (2020). Access to the data will be made available upon completion of the entire project and is available upon reasonable request from the corresponding author.
