# Supplementary material for: The mysterious case of the disappearing pilot study: a review of publication bias in preliminary behavioral interventions presented at health behavior conferences
Source: Pilot Feasibility Stud. 2023 Jul 7;9:115. doi: 10.1186/s40814-023-01345-8 (PMC10327298; doi:10.1186/s40814-023-01345-8)
Supplement: Supplementary file 1 — Additional file 1: Appendix 1. Survey Questions. [file 40814_2023_1345_MOESM1_ESM.pdf]

Appendix 1: Survey

Start of Block: Block 1

Q1.1 Dear Author,

My name is Michael Beets. I am a professor in the Department of Exercise Science at the University of South Carolina. We are conducting a research study and I would like to invite you to participate. This study is funded by the National Heart, Lung, and Blood Institute at the NIH and has been approved by the UofSC IRB (Pro00101406).

You are receiving this survey because you can provide valuable insight into the reasons why some pilot/feasibility studies remain unpublished.   It will take you approximately **8 minutes to complete this surve**y comprised of several multiple choice questions.

Once you complete the survey, you will have an opportunity to enter your email address to receive a **$25 Amazon gift card**. Your answers will remain anonymous.   We will be happy to answer any questions you have about the study. You may contact me at beets@mailbox.sc.edu.

Thank you for your consideration. If you would like to participate, please select an option below and continue onto the survey.

With kind regards,

Michael W. Beets
Arnold School of Public Health
University of South Carolina
(803) 777-3003
beets@mailbox.sc.edu

Q1.2 Please select whether you would like to participate in this study.

- ◯ **YES** - I have read the study information above and agree to participate in this study
- ◯ **NO** - I do not agree to participate in this study

End of Block: Block 1

Start of Block: Survey Questions

Q2.1 What is your age?

- ◯ 18-24 years old
- ◯ 25-34 years old
- ◯ 35-44 years old
- ◯ 45-54 years old
- ◯ 55-64 years old
- ◯ 65-74 years old
- ◯ 75 years or older

Q2.2 Which best describes your gender?

- ◯ Male
- ◯ Female
- ◯ Non-binary / third gender
- ◯ Prefer not to say
- ◯ Write-in ____

Q2.3 In which country do you currently reside?
Q2.4 The follow questions refer to a study presented in ${e://Field/year}
at ${e://Field/conference} titled ${e://Field/title}

Q2.5 Thinking back to the study listed above, are any of the following true?
[*Please select all that apply*]

- ☐ This study was published as a full-length article in a peer-reviewed journal.
- ☐ Data produced by this study was intended for use in a deliverable other than a peer-reviewed publication (e.g., government report, program evaluation, product for a community partner).
- ☐ Study sponsor or community partner did not permit publication in a peer-reviewed journal.
- ☐ Use of study findings were at the discretion of a commercial or government entity.
- ☐ This study was conducted as part of a doctoral dissertation, master's thesis, or other student-led academic project.
- ☐ None of the above

Q2.6 You indicated that this study was published as a full-length article in a peer-reviewed journal. Could you please provide the citation, URL, or DOI for this publication?

Q2.7 When conducting the previously mentioned study, did you initially intend to publish your findings in a peer-reviewed journal?

- Yes
- No
- I don't remember

Q2.8 Did you ever submit this study for publication in a peer-reviewed journal?

- Yes
- No
- I don't remember

Q2.9 Did you ever use any data from this study to support a grant application?

- Yes
- No
- I don't remember

Page Break

Q2.10 When attempting to publish this study, how many journals did you submit your study to?

- ◯ 1
- ◯ 2
- ◯ 3
- ◯ 4 or more
- ◯ I don't remember
- ◯ Not applicable

Q2.11 How many grant applications did you submit that included data produced by this study? [*Please count all grant application* including internal/intramural and external]

- ◯ 1
- ◯ 2
- ◯ 3
- ◯ 4 or more
- ◯ I don't remember
- ◯ Not applicable

Page Break

Q2.12 Pilot studies may not be published due to study characteristics. Are any of the following reasons that your study was not published?[Please select all that apply]

- ☐ Small or insignificant effects
- ☐ Negative results
- ☐ Null Results
- ☐ Unexpected results
- ☐ Results were unimportant
- ☐ Your other publications had similar findings
- ☐ Publications by other authors had similar findings
- ☐ Limitations in methodology
- ☐ Inability to collect sufficient data
- ☐ None of the above
- ☐ Other study characteristics:

Q2.13 Pilot studies may not be published due to **external factors**.
Are any of the following reasons that your study was not published?

[*Please select all that apply*]

- ☐ Difficulties with co-authors
- ☐ I relocated or retired
- ☐ Project was student led and the student left or graduated
- ☐ Project was led by a staff member that left or graduated
- ☐ Project was led by a faculty member that left or retired
- ☐ Funding cycle for the project/study ended
- ☐ Unable to address reviewer comments
- ☐ Other projects/studies took priority
- ☐ None of the above
- ☐ Other external factors: ____________________

Q2.14 Pilot studies may not be published due to resources. Are any of the following reasons that your study was not published[Please select all that apply]

- ☐ Lack of money for publication fees
- ☐ Lack of money for open access fees
- ☐ Lack of time to write
- ☐ Lack of administrative support at institution
- ☐ Lack of access to English-language support
- ☐ Funding for another project redirected my time/attention
- ☐ None of the above
- ☐ Other resources: ____

Q2.15 Pilot studies may not be published due to **features common in pilot studies**.
Are any of the following were reasons that your study was not published?

[*Please select all that apply*]

- ☐ Small sample size
- ☐ Did not test outcomes for statistical significance
- ☐ Short study duration
- ☐ Intervention delivered by PI
- ☐ Intervention delivered by student
- ☐ Lack of high-quality measures
- ☐ None of the above
- ☐ Other pilot study features: ____________________

Q2.16 Have you ever been a first or lead author on a published pilot/feasibility behavioral intervention?
[*please only include full-length, peer-reviewed publications*]

- ○ Yes
- ○ No
- ○ Unsure

Q2.17 Compared to non-pilot behavior interventions, how difficult is it to publish a pilot or feasibility study?

- ○ Extremely difficult
- ○ Somewhat difficult
- ○ Neither easy nor difficult
- ○ Somewhat easy
- ○ Extremely easy

Q2.18 Are you familiar with non-traditional outlets for research products (e.g., Research Gate, Octopus, Open Science Forum)?
[*Please do not include data repositories*]

- ○ Yes
- ○ No

Q2.19 Have you ever used non-traditional outlets to share your research products?
[P*lease do not include Twitter or other social media outlets]*

- ○ Yes
- ○ No

Q2.20 Do you have any additional thoughts on publishing pilot behavioral intervention studies that you would like to share?

____

End of Block: Survey Questions

Start of Block: Gift Card Opt In

Q3.1 Would you like to provide your email address to receive a $25 Amazon gift card?
Your responses will still remain anonymous.

- ◯ Yes
- ◯ No

End of Block: Gift Card Opt In
