# Supplementary material for: The mysterious case of the disappearing pilot study: a review of publication bias in preliminary behavioral interventions presented at health behavior conferences
Source: Pilot Feasibility Stud. 2023 Jul 7;9:115. doi: 10.1186/s40814-023-01345-8 (PMC10327298; doi:10.1186/s40814-023-01345-8)
Supplement: Supplementary file 2 — Additional file 2: Appendix 2. Open Ended Responses. [file 40814_2023_1345_MOESM2_ESM.docx]

**Appendix 2: Open Ended Responses**

**Survey Question (Q2.20):**

Do you have any additional thoughts on publishing pilot behavioral intervention studies that you would like to share?

**Survey Responses:**

Pilot studies have difficulty being published because are viewed as not being as scientifically rigorous as full-scale trials and there are few journals dedicated to publishing pilot work. Distinguishing between pilot and feasibility studies may also be an issue. – ID40

Brief reports have proven to be a useful outlet for publishing pilot findings; we've also tried publishing letters to the editor of relevant journals. – ID 36

I just want to clarify that while I correctly identified some limiting factors to our publication, this manuscript is still currently under review and we are still seeking to publish it. – ID37

It is difficult for non-doctoral level researchers to have adequate mentorship and/or support from senior staff to lead author manuscripts. – ID39

The field, as a whole, needs more education about what a pilot is and what it isn't. We tend to do "feasibility and preliminary efficacy studies," but we recognize it is not really efficacy that we're powered to assess. – ID41

I think sharing pilot study information can be very important as it will provide opportunities for other investigators to have pilot information/data before collaborating on a larger study. – ID38

There is a home for everything and just being honest that it is a pilot feasibility and focusing on treatment fidelity helps. -ID42

**Survey Question (Q2.12):**

Pilot studies may not be published due to study characteristics. Are any of the following reasons that your study was not published? Open text option:

**Survey Responses:**

Please note this study was published as a brief report (not a full length journal article)--but only recently and may not have been published at the time you did your research. – ID34

Very small sample size -ID35

Very small sample size – ID38

Relatively small sample size – ID 43

**Survey Question(Q2.13):**

Pilot studies may not be published due to external factors. Are any of the following reasons that your study was not published? Open text option:

**Survey Responses:**

Not yet had the time – ID33

Publication was delayed due to relocation – ID 34

The manuscript was being written by a student who left after the manuscript had received 2-3 reviews. – ID40

As a PI without a research coordinator and multiple funded studies, this study in particular has been put on a back-burner. Also, the timing of this study was problematic wherein student assistants have come and gone and its difficult to get new students potentially interested in these data, up to speed on the protocol, data, etc. – ID41

**Survey Question(Q2.14):**

Pilot studies may not be published due to resources. Are any of the following reasons that your study was not published? Open text option:

**Survey Responses:**

Lack of expertise to write the full paper – ID32

Student assistance turnover has been high in the past 4-5 years. – ID41

**Survey Question(Q2.15):**

Pilot studies may not be published due to features common in pilot studies. Are any of the following were reasons that your study was not published? Open text option:

**Survey Responses:**

PI left the organization - ID38
